# Universal Access to Xpert MTB/RIF Testing for Diagnosis of Tuberculosis in Uzbekistan: How Well Are We Doing?

**DOI:** 10.3390/ijerph18062915

**Published:** 2021-03-12

**Authors:** Laziz Turaev, Ajay Kumar, Dilyara Nabirova, Sevak Alaverdyan, Nargiza Parpieva, Barno Abdusamatova

**Affiliations:** 1 National Reference Laboratory, Tashkent 100086, Uzbekistan; 2 International Union Against Tuberculosis and Lung Disease, South-East Asia Office, New Delhi 110016, India; akumar@theunion.org; 3 International Union Against Tuberculosis and Lung Disease, 75006 Paris, France; 4 Yenepoya Medical College, Yenepoya (Deemed to be University), Mangaluru 575018, India; 5 United States Centers for Disease Control and Prevention, Central Asia Region, Almaty A25T0A1, Kazakhstan; hny5@cdc.gov; 6 Bielefeld Graduate School of Economics and Management (BiGSEM), Bielefeld University, Universitätsstraße, 33615 Bielefeld, Germany; s.alaverdyan@iset.ge; 7 National Tuberculosis Programme, Ministry of Health, Tashkent 100086, Uzbekistan; nargizaparpieva@gmail.com; 8 Ministry of Health, Tashkent 100086, Uzbekistan; barno.abdusamatova@minzdrav.uz

**Keywords:** nucleic acid amplification tests, polymerase chain reaction, rapid molecular diagnostics, central Asia, missing cases, operational research, SORT IT, pre-diagnosis attrition

## Abstract

As per national guidelines in Uzbekistan, all presumptive tuberculosis patients should be tested using the Xpert MTB/RIF assay for diagnosing tuberculosis. There is no published evidence how well this is being implemented. In this paper, we report on the Xpert coverage among presumptive tuberculosis patients in 2018 and 2019, factors associated with non-testing and delays involved. Analysis of national aggregate data indicated that Xpert testing increased from 24% in 2018 to 46% in 2019, with variation among the regions: 21% in Tashkent region to 100% in Karakalpakstan. In a cohort (January–March 2019) constituted of 40 randomly selected health facilities in Tashkent city and Bukhara region, there were 1940 patients of whom 832 (43%, 95% confidence interval (CI): 41–45%) were not Xpert-tested. Non-testing was significantly higher in Bukhara region (73%) compared to Tashkent city (28%). In multivariable analysis, patient’s age, distance between primary health centre (PHC) and Xpert laboratory, diagnostic capacity and site of PHC were associated with non-testing. The median (interquartile range) duration from date of initial visit to PHC to receiving results was 1 (1–2) day in Tashkent city compared to 3 (1–6) days in Bukhara region (*p*-value < 0.001). While there is commendable progress, universal access to Xpert testing is not a reality yet.

## 1. Introduction

Globally, an estimated 10 million people developed tuberculosis (TB) in 2019, of whom, only 7.1 million were reported by countries. This leaves a gap of 29%, and such patients are referred to as the ‘missing millions’ [1]. These missed cases include the following: (i) patients who did not access health care; (ii) patients who reached a health facility, but were not identified as having presumptive TB (previously known as TB suspects) by the health care providers; (iii) patients who were identified as ‘presumptive TB’, but were not investigated with the correct tests and diagnosed; (iv) patients who were diagnosed, but not started on treatment, and (v) patients who were treated (especially in the private health sector), but not reported to the national TB programmes (NTP) and the World Health Organization (WHO) [2].

Unless we are able to identify these ‘missing’ millions and place them on appropriate treatment, we will not be able to reach the targets envisioned in the WHO’s End TB strategy and United Nation’s Sustainable Development Goals (SDGs)—which are 95% reduction in TB deaths, and a 90% reduction in TB incidence by 2035, compared with 2015 [3,4]. WHO’s global plan to end TB envisions 90-(90)-90 targets which equate to detecting 90% of all TB cases (including 90% among key populations) and successfully treating 90% of all diagnosed cases [5].

The gaps in diagnosis and treatment are wider for drug-resistant TB as compared to drug-susceptible TB. In 2019, there were an estimated 465,000 people who developed TB that was resistant to rifampicin (RR-TB), and of these, 78% had multidrug-resistant TB (MDR-TB, defined as resistance to both isoniazid and rifampicin). A total of 201,938 (43%) patients were diagnosed and of these, 177,091 (88%) were started on treatment. This indicates that the major gap is in diagnosis of MDR/RR-TB [1]. To address this, WHO recommends using the Xpert MTB/RIF assay as the primary diagnostic test of choice among people with presumptive TB, especially in settings with a high burden of MDR-TB.

Uzbekistan is one such country and features among the top 30 high-burden MDR-TB countries in the world [6]. A national drug resistance survey conducted in 2010–2011 estimated that 23% of new patients and 62% of previously treated TB patients had MDR-TB [7]. The WHO estimates that 3200 patients developed MDR/RR-TB in 2019 in Uzbekistan. Of these, only 2060 (64%) were diagnosed. This means about one-third of the MDR/RR-TB patients remain undiagnosed transmitting the disease in the community. To address this, the Ministry of Health of Uzbekistan has embarked on rapid TB diagnosis since 2012 using Xpert MTB/RIF^®^ assay (Cepheid Inc., Sunnyvale, CA, USA) that detects both TB and rifampicin resistance.

As per the NTP guidelines, all presumptive TB patients are expected to be routinely tested using Xpert MTB/RIF assay as the first diagnostic of choice. Presumptive TB patients visiting the primary healthcare centers (PHC) are expected to be referred to (or their sputum samples transported) to the nearest health facility having Xpert MTB/RIF testing services. The access to Xpert MTB/RIF testing has been expanded and the number of GeneXpert machines has almost doubled from 35 in 2018 to 67 in 2019 (Table 1). Despite this scale-up, anecdotal evidence indicates that there may be gaps in Xpert MTB/RIF testing.

Globally, there is limited information about the gap between ‘being identified as presumptive TB’ and ‘getting tested’ with TB diagnostic tests. A few studies from Asia and Africa indicate high rates of non-testing among both presumptive drug-sensitive and presumptive drug-resistant TB patients [8,9,10,11,12,13,14]. However, there is no published evidence on this issue from Uzbekistan. Quantifying the extent of Xpert MTB/RIF testing coverage and its associated factors and finding out the delays in testing may help the NTP to make appropriate changes in programme policy and practice. Hence, we conducted this operational research study with the aim of assessing the Xpert MTB/RIF test coverage among people with presumptive TB, factors associated with it and the delays involved. The specific objectives were:

1. To determine nationally in Uzbekistan, and stratified by region, for the years 2018 and 2019:
  - The aggregate number of presumptive TB patients;
  - The aggregate number (proportion) tested using Xpert MTB/RIF assay.
2. In an individual patient-wise cohort of presumptive TB patients identified in selected health care facilities of Tashkent City and Bukhara Region during January–March 2019, to determine:
  - The number (proportion) tested using Xpert MTB/RIF and/or microscopy and the number (proportion) diagnosed with TB;
  - Demographic and health-facility level factors associated with not getting tested using Xpert MTB/RIF assay;
  - Median duration in days between the ‘date of initial visit’ to the PHC and ‘date of PHC receiving the Xpert MTB/RIF result’.

## 2. Materials and Methods

### 2.1. Study Design

For objective 1, we used an ecologic study design using aggregate data collected from the regions and compiled at the national level. For objective 2, we used a cohort study design involving secondary analysis of routine programme data from Tashkent City and Bukhara region.

### 2.2. Setting

Uzbekistan is a double land-locked country in Central Asia (part of the former Soviet Union) with an estimated population of 33 million people. Uzbekistan is a lower-middle income country and consists of 12 regions (or oblasts), one autonomous republic (the Republic of Karakalpakstan), and Tashkent metropolitan area, the capital city [15]. The regions are further divided into districts (tumans) and cities. There are 199 districts or cities in the country.

#### 2.2.1. Tuberculosis (TB) Control Program in Uzbekistan

TB control activities are coordinated countrywide by the NTP based out of Republican Specialized Scientific Practical Medical Centers of Phthisiology and Pulmonology. TB diagnosis and treatment are provided free of charge within the NTP. There are no private TB services. All the drugs used in the NTP are quality-assured and procured through the support of the Global Fund as well as through the republican budget.

In each province, as well as in Tashkent city and the Republic of Karakalpakstan, there are provincial dispensaries that implement oversight of TB control at local level. District level facilities are represented either as district TB dispensaries or as TB departments within central district hospitals. They oversee the implementation of TB services and link PHCs with the provincial TB dispensaries. There are 3437 health care facilities in the country in both urban and rural areas. Presumptive TB patients are identified at these facilities and referred to the specialized TB facilities for diagnosis and treatment. PHCs are also involved in providing treatment to patients during the continuation phase of treatment. Case finding, diagnosis, treatment regimens, treatment outcomes and monitoring and evaluation follow the WHO guidelines.

#### 2.2.2. The TB Laboratory Network

The TB laboratory network in Uzbekistan comprises sputum smear microscopy laboratories, GeneXpert laboratories, eight line probe assay (LPA) laboratories, and seven laboratories which can perform all types of diagnostic tests including culture and drug susceptibility testing (DST). The laboratories are classified into five tiers as follows:

- Tier I—Sputum smear microscopy laboratories without GeneXpert, situated at district level dispensaries and PHCs;
- Tier II—Laboratories with both microscopy and GeneXpert situated at provincial, district level or PHCs;
- Tier III—Provincial level laboratories capable of performing LPA;
- Tier IV—Inter-provincial level laboratories which conduct LPA and culture and DST;
- Tier V—National reference laboratories which conduct LPA and culture and DST.

#### 2.2.3. Recording and Reporting

A health worker registers each patient in the sputum collection logbook and fills out a referral form for testing indicating the patient’s name, date of birth, address, test purpose (diagnosis or follow-up) and sample collection date. The sputum sample is then collected and sent along with the form to the designated laboratory. Sputum samples from PHCs are transported to the GeneXpert laboratories using the vehicles owned by PHCs during planned working visits at-least once a week. Xpert MTB/RIF test details and results are recorded in a laboratory register, which is then captured in an electronic database (MS Access) and submitted to the national level by email, once a month. The results are communicated back to the PHCs when the vehicle visits the GeneXpert laboratory the next time. It is recommended that the time from sputum collection to declaring the results should not exceed 48 h.

### 2.3. Study Population

For the first objective, we included all presumptive TB patients in Uzbekistan during 2018 and 2019, except those attending human immunodeficiency virus (HIV) care facilities and prison health facilities. ‘Presumptive TB’ refers to a patient who presents with symptoms or signs suggestive of TB (previously known as TB suspect) and documented in the sputum collection logbook maintained at the health facilities.

For the second objective, we included all presumptive TB patients visiting the selected health facilities in Tashkent City and Bukhara Region between January and March 2019. There are 182 healthcare facilities in Tashkent City. Of them, 29 conduct sputum smear microscopy including four which also conduct the Xpert MTB/RIF assays. Of the 288 health facilities in Bukhara region, 15 facilities conduct sputum smear microscopy including four with Xpert MTB/RIF testing. We selected a random sample of 20 health facilities each from Tashkent city and Bukhara region for this study—in such a way that there is adequate representation of the health care facilities with different diagnostic capacities ((i) those with GeneXpert (ii) microscopy only and (iii) neither microscopy nor GeneXpert). Thus, a total of 40 health facilities were selected. Data were collected between January and May 2020.

### 2.4. Data Variables and Sources

For objective 1, the Ministry of Health sent out a communication to all the Regional TB hospitals and dispensaries to collect data on required variables from each of the health facilities in their respective region. This was then compiled at the national level. Data on numbers tested were also extracted from the national laboratory database to validate the data provided by the regions. In case of discrepancies, we considered the numbers extracted from laboratory database as final.

For objective 2, we first visited each selected health facility and made a digital copy of the sputum collection log book using MS Excel (database 1). We then extracted the data from the Xpert laboratory database (database 2). Then, we merged the two databases using ‘Fuzzy Lookup’ add-in (which is free and helps in controlling the extent of matching) in MS Excel and created a master dataset for analysis. Since there was no unique identification number linking the two databases, we used name, age and sex as the primary identifiers. We considered a match only if there was a match with name (minor spelling errors were ignored), exact match with sex and approximate match with age (plus or minus 3 years). Patients without a match in the laboratory database using the criteria mentioned above were considered as ‘not tested’ for Xpert MTB/RIF.

The key exposure variables included age, sex, region, site of health facility (whether centrally located in the city or peripherally located in rural areas), diagnostic capacity (both Xpert and microscopy, only microscopy or ‘neither microscopy nor Xpert’) and distance between the ‘health facility visited by the presumptive TB patient’ and the ‘nearest facility providing Xpert MTB/RIF testing services’. We also collected the dates of initial patient visit, date of sputum collection, date of sputum receipt at Xpert laboratory and date of receipt of results to assess the delays involved in the diagnostic process.

### 2.5. Analysis and Statistics

Data were analyzed using EpiData Analysis (version 2.2.2.187, EpiData Association, Odense, Denmark) and Stata software (version 16.0, Statacorp, TX, USA). Demographic and clinical characteristics were summarized as percentages. Delays in testing were summarized using median and interquartile ranges (IQR). A chi-square test or Fischer’s exact test was used as appropriate to compare the proportions. The Kruskal–Wallis test was used to test the difference between medians. The key outcome was ‘not getting tested using Xpert MTB/RIF assay’. Unadjusted relative risks with 95% confidence intervals (CI) were calculated to assess the associations between demographic and health-facility level factors and Xpert MTB/RIF non-testing. A multivariable model (Poisson regression with robust standard errors) was used and adjusted relative risks with 95% CI were deduced [16,17,18]. All the variables with a *p* value of <0.2 in unadjusted analysis were included in the regression model. Since ‘region’ and ‘site of health facility’ were collinear, we included the latter only in the final model. Model fit was tested using goodness-of-fit chi-square test.

## 3. Results

### 3.1. National and Regional Xpert MTB/RIF Test Coverage

**Figure 1:**
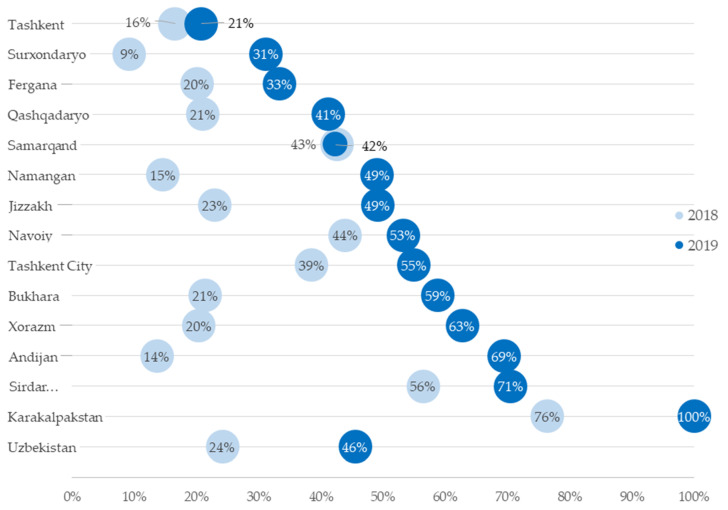
Change in Xpert MTB/RIF test coverage among presumptive tuberculosis patients visiting health care facilities of Uzbekistan from 2018 (light blue circles) to 2019 (dark blue circles).

Analysis of aggregate data indicated that Xpert MTB/RIF test coverage among presumptive TB patients increased from 24% in 2018 to 46% in 2019 (Table 2). While there was an increase in test coverage in all the regions of the country, the extent of increase was variable (Figure 1). In 2019, there was huge variation in Xpert MTB/RIF test coverage among the regions—from 21% in Tashkent region (outside the city) to 100% in the republic of Karakalpakstan.

### 3.2. Baseline Characteristics

There were 1940 presumptive TB patients in our study sample, of whom 1282 were from Tashkent city and 658 were from Bukhara region. The baseline characteristics of these patients are shown in Table 3. Among the 1940 patients, 918 (47%) were females and the mean age was 52 (standard deviation: 18) years. There was a significant difference between Tashkent and Bukhara with respect to the sex distribution of patients, distance from PHC of initial visit of the patient to the nearest GeneXpert laboratory and the diagnostic capacity of facilities. The median distance from the PHC of initial visit to the nearest GeneXpert laboratory was 15 km (IQR 11–17) in Tashkent city and 17 km (IQR 10–40) in Bukhara region (*p* < 0.001). About 51% (332/658) of the patients in Bukhara region visited a health facility with no diagnostic services, while this was only 9% (110/1282) in Tashkent city. Similarly, while nearly 80% of patients of Bukhara region visited peripherally located rural PHCs, 88% of patients of Tashkent city were from centrally located health facilities.

### 3.3. Diagnosis of Tuberculosis by Test Used

**Figure 2:**
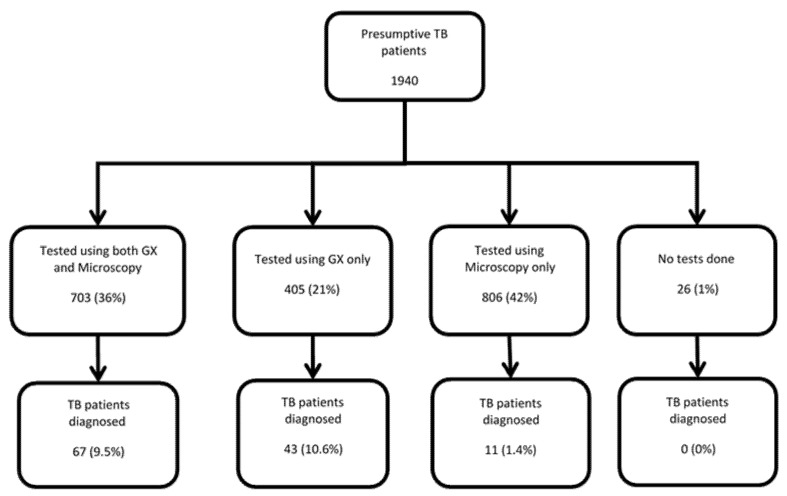
Presumptive TB patients tested using Xpert MTB/RIF assay and/or sputum smear microscopy and TB patients detected in selected health care facilities of Tashkent city and Bukhara regions of Uzbekistan, January–March 2019; TB = tuberculosis; RR = rifampicin resistance; GX = GeneXpert.

Of the 1940 patients, only 26 (1%) did not receive any diagnostic services. The remaining 99% of the patients were tested using either microscopy (806, 42%) or Xpert MTB/RIF (405, 21%) or both (703, 36%) (Figure 2). A total of 121 (6.2%) TB patients were diagnosed, of whom 34 (28%) had rifampicin resistance. TB positivity was 9.5% among patients tested with ‘both microscopy and Xpert MTB/RIF’, 10.6% among patients tested using ‘Xpert MTB/RIF only’ and 1.4% among patients tested with ‘microscopy only’.

### 3.4. Factors Associated with Xpert MTB/RIF Non-Testing

Of the 1940 patients, 832 (43%, 95% CI: 41–45%) did not undergo Xpert MTB/RIF testing. Non-testing was significantly higher in Bukhara region (73%, 95% CI: 69–76%) compared to Tashkent city (28%, 95% CI: 25–30%) (*p* value < 0.001). In multivariable analysis, age, distance, diagnostic capacity and site of the health facility were associated with Xpert MTB/RIF non-testing (Table 4). Compared to patients aged 15–34 years, other age groups had a higher risk of non-testing. Patients visiting a PHC located at a distance of 10 kilometres or more from the GeneXpert laboratory had a higher risk of non-testing compared to a distance of <10 km. Patients visiting a health facility with no diagnostic services had the highest risk of non-testing followed by those visiting a health facility with ‘only microscopy’ services as compared to those visiting a health facility with Xpert MTB/RIF testing services.

### 3.5. Turnaround Time of Laboratory Results

The times taken at different steps of the cascade are shown in Table 5. The median (IQR) duration from date of initial visit to receiving the laboratory result was 1 (1–2) day in Tashkent city compared to 3 (1–6) days in Bukhara region (*p* value < 0.001).

## 4. Discussion

This is the first study from Uzbekistan assessing the Xpert MTB/RIF test coverage among presumptive TB patients, factors associated with non-testing and time taken to obtain results.

There has been great progress in Xpert MTB/RIF testing across the country—test coverage almost doubled in 2019 compared to 2018 and this was accompanied by the doubling of GeneXpert machines in the country. However, about half of all presumptive TB patients in 2019 did not receive Xpert MTB/RIF testing. Also, the test coverage varied hugely across the regions with the highest coverage of 100% in Karakalpakstan and the lowest coverage in Tashkent region, where only one in five patients was tested. So, there is a long way to go before the dream of universal access to rapid molecular diagnostics for TB is realized in Uzbekistan.

While the exact reasons for the inter-regional variation are unknown, we speculate on some of these based on our programmatic experience. The excellent performance in Karakalpakstan may be explained by several initiatives from Médecins Sans Frontières (MSF), which has been providing both technical and financial support to the NTP for over two decades [19]. These initiatives include capacity building of health care providers and provision of performance-based incentives to them, an excellent system for sputum collection and transport from all PHCs to GeneXpert laboratories, and a strong procurement and supply chain management system which ensures that the necessary equipment (including annual recalibration and maintenance of the machines) and laboratory supplies are available all the time. This system is supported by regular supportive supervision and monitoring visits by MSF.

By contrast, all the other regions in the country had suboptimal test coverage. This may be due to many reasons. Sample collection and transport systems are not optimal in many places with samples being transported in PHCs’ own vehicles once or twice a week. Rarely, patients are referred to GeneXpert laboratories and they may not reach the center especially in peripheral and rural areas. Sometimes, GeneXpert machines breakdown due to lack of annual calibration and maintenance. There may also be gaps in the uninterrupted supply of test cartridges (often due to lengthy custom clearances) which may contribute to non-testing of presumptive TB patients [20,21]. Lastly, the health care providers may not be aware of the new diagnostic algorithms and may still be relying on microscopy. This is especially so in places with a high turnover of health care providers. This needs to be addressed through periodic training.

We found several factors associated with non-testing. First, distance was a major factor. Patients visiting PHCs situated far away from the GeneXpert laboratories had a higher risk of non-testing. Shorter distances might explain why delays in testing were less in Tashkent city when compared to Bukhara region. Second, the presence of diagnostic service at the same facility was a major determinant of testing. Patients visiting PHCs with no diagnostic services had the highest risk of non-testing.

A positive finding of this study was that only 1% of presumptive TB patients did not receive any diagnostic evaluation. However, the yield of TB among those who received ‘microscopy only’ was low at 1.4% as compared to ~10% in those receiving Xpert MTB/RIF testing—suggesting that a substantial proportion of patients (including those with rifampicin resistance) are missed (or diagnosis and treatment delayed) when GeneXpert is not used for diagnosis. This provides strong evidence about the crucial role of Xpert MTB/RIF testing in tackling the epidemic of drug-resistant TB in Uzbekistan.

Our study had several strengths. This is the first time that aggregate data have been compiled and used to provide national and regional estimates of Xpert test coverage. We had a large sample in the cohort study which helped in performing a robust analysis of factors associated with non-testing. The sample had representation from both urban and rural regions and thus the findings may be generalizable to other similar settings in the country. Since we used routine programme data for our analysis, it reflects the true situation on the ground.

There were some limitations too. We could not obtain data from HIV care facilities and prison health facilities, and hence our findings do not reflect the situation there. This needs future research. We were limited by the variables available in the routine records, and hence we may have missed other important factors related to non-testing. We also did not interview the patients or providers to understand the reasons for non-testing from their perspective. This requires further investigation using qualitative research methods. Since there was no identification number linking the sputum collection logbook at PHCs to the laboratory register at the GeneXpert laboratory, we used name, age and sex to identify a match. There may have been errors in this process and we may have underestimated the overall levels of Xpert testing.

There are several implications of the study findings. First, we recommend continued scale-up of GeneXpert machines so that there is one machine on average for every district in the country. This will improve access by reducing the distances to be covered during sample transport, although this means more machines that need maintenance, a further need for trained personnel and stronger systems of supply chain management of test cartridges [20,21]. To enable optimization, a systematic assessment of utilization of existing GeneXpert machines needs to be undertaken [22]. Second, sample collection and transport systems from the PHC to the GeneXpert laboratory need to be strengthened. A cost-effectiveness assessment of scaling up GeneXpert machines vis-à-vis strengthening sample collection and transport needs to be undertaken to inform the way forward. Third, digital laboratory surveillance systems such as GxAlert should be established to enable real-time sharing of test results with patients and providers [23]. Such systems will also help in performing a cohort analysis periodically and assess progress. However, this requires many steps such as strengthening the digital infrastructure (computer and internet) at PHCs and improving computer literacy among the health care providers. Fourth, the capacity of health care providers needs to be built using induction and refresher training so that they can use the new diagnostic algorithms requiring universal testing of presumptive TB patients using Xpert MTB/RIF assay. The experience of Karakalpakstan may be adopted by other regions with annually allocated resources for health care provider training, sample storage, transportation and processing, development of the online laboratory information systems with the same unique identifier at PHCs and reference laboratories and efficient sharing of results in real-time [24]. Finally, we recommend inclusion of simple indicators such as ‘number of presumptive TB patients visiting the PHC’ and ‘number tested using Xpert MTB/RIF’ in the monthly reports. This may be analysed to monitor the performance at national, regional, district and health facility levels.

## 5. Conclusions

In conclusion, nearly half of all presumptive TB patients in 2019 in Uzbekistan received Xpert MTB/RIF testing. While this is commendable progress compared to 2018, it is still a long way to reach universal access to care. We found several factors associated with non-testing which include distance of the PHC from the GeneXpert laboratory, diagnostic capacity of the PHC and location of the PHC. Future assessments should focus on assessing the reasons for the gaps using qualitative research methods.

## Figures and Tables

**Table 1:** Availability of GeneXpert machines in health facilities of Uzbekistan in 2018 and 2019.

Province or City	Number of GeneXpert Machines in 2018	Number of GeneXpert Machines in 2019	Number of Districts or Cities	Districts with GeneXpert (%)
Andijan	3	4	17	24%
Bukhara	1	4	13	31%
Fergana	2	4	19	21%
Jizzakh	1	4	13	31%
Karakalpakstan	6	8	16	50%
Namangan	3	5	12	42%
Navoiy	1	3	10	30%
Qashqadaryo	1	4	15	27%
Samarqand	2	4	16	25%
Sirdaryo	1	3	11	27%
Surxondaryo	2	3	14	21%
Tashkent	2	4	19	21%
Tashkent City	2	4	11	36%
Xorazm	1	3	13	23%
National	28 *	57 **	199	29%

* In addition, there were 2 GeneXpert machines in national reference laboratories, 3 in human immunodeficiency virus (HIV) care facilities and 2 in prisons in 2018—thus a total of 35 machines in the country. ** In addition, there were 2 GeneXpert machines in national reference laboratories, 5 in HIV care facilities and 3 machines in prisons in 2019—thus a total of 67 machines in the country.

**Table 2:** Xpert MTB/RIF test coverage among presumptive tuberculosis (TB) patients visiting the health care facilities of Uzbekistan in 2018 and 2019 *.

Province/Region	2018			2019		
	Number of Presumptive TB Patients *	Xpert MTB/RIF Testing		Number of Presumptive TB Patients *	Xpert MTB/RIF Testing	
	N	*n*	(%)	N	*n*	(%)
Andijan	19,419	2641	(14)	9973	6929	(69)
Bukhara	22,109	4731	(21)	20,897	12,290	(59)
Fergana	25,540	5114	(20)	25,162	8391	(33)
Jizzakh	11,685	2678	(23)	11,499	5646	(49)
Karakalpakstan	15,614	11,926	(76)	11,462	11,462	(100)
Namangan	26,280	3835	(15)	13,819	6774	(49)
Navoiy	5670	2490	(44)	9656	5138	(53)
Qashqadaryo	15,371	3230	(21)	17,115	7049	(41)
Samarqand	9737	4151	(43)	16,813	7124	(42)
Sirdaryo	5206	2937	(56)	6406	4517	(71)
Surxondaryo	32,637	3000	(9)	15,164	4726	(31)
Tashkent	25,592	4217	(16)	40,979	8512	(21)
Tashkent City	9054	3487	(39)	8722	4791	(55)
Xorazm	10,835	2202	(20)	6667	4181	(63)
National	23,4749	56,639	(24)	214,334	97,530	(46)

* This does not include the presumptive TB patients attending the prison health facilities and HIV care facilities.

**Table 3:** Baseline characteristics of presumptive tuberculosis patients in selected health care facilities of Tashkent and Bukhara regions of Uzbekistan, January–March 2019.

Characteristics	Tashkent		Bukhara		Total	
	N	%	N	%	N	%
**Total**	1282	(100)	658	(100)	1940	(100)
**Age (Years)**						
Less than 15	17	(1.3)	3	(0.5)	20	(1.0)
15–34	293	(22.9)	66	(10.0)	359	(18.5)
35–54	444	(34.6)	173	(26.3)	617	(31.8)
55–74	461	(36.0)	308	(46.8)	769	(39.6)
75 and above	67	(5.2)	108	(16.4)	175	(9.0)
**Sex**						
Male	738	(57.6)	284	(43.2)	1022	(52.7)
Female	544	(42.4)	374	(56.8)	918	(47.3)
**Site of Health Facility**						
Tashkent peripheral	159	(12.4)	0	(0.0)	159	(8.2)
Tashkent central	1123	(87.6)	0	(0.0)	1123	(57.9)
Bukhara peripheral	0	(0.0)	524	(79.6)	524	(27.0)
Bukhara central	0	(0.0)	134	(20.4)	134	(6.9)
**Diagnostic Capacity**						
Microscopy and GeneXpert	452	(35.3)	103	(15.7)	555	(28.6)
Microscopy only	720	(56.2)	223	(33.9)	943	(48.6)
No microscopy no GeneXpert	110	(8.6)	332	(50.5)	442	(22.8)
Distance (km) *						
0–9	652	(50.9)	240	(36.5)	892	(46.0)
10–19	553	(43.1)	167	(25.4)	720	(37.1)
20 and above	77	(6.0)	251	(38.1)	328	(16.9)

* Distance from the health facility where the patient initially visited to the nearest GeneXpert laboratory; km = kilometers.

**Table 4:** Factors associated with not getting tested using the Xpert MTB/RIF assay among presumptive tuberculosis patients in selected health care facilities of Tashkent city and Bukhara region of Uzbekistan, January–March 2019.

	Total	Not Tested Using Xpert		RR	(95% CI)	aRR	(95% CI)
		N	(%)				
**Total**	1940	832	(43)				
**Sex**							
Male	1022	402	(39)	Ref.		Ref.	
Female	918	430	(47)	1.19	(1.07,1.32)	1.04	(0.96,1.12)
**Age (Years)**							
Less than 15	20	8	(40)	1.71	(0.97,3.02)	1.74	(1.09,2.77)
15–34	359	84	(23)	Ref.		Ref.	
35–54	617	241	(39)	1.67	(1.35,2.06)	1.35	(1.15,1.59)
55–74	769	390	(51)	2.17	(1.77,2.65)	1.46	(1.25,1.71)
75 and above	175	109	(62)	2.66	(2.14,3.32)	1.45	(1.22,1.73)
Distance (km) *							
0–9	892	232	(26)	Ref.		Ref.	
10–19	720	359	(50)	1.92	(1.68,2.19)	1.66	(1.45,1.88)
20 and above	328	241	(73)	2.83	(2.48,3.21)	1.77	(1.52,2.05)
**Site of Health Facility**							
Tashkent central	1123	203	(18)	Ref.		Ref.	
Tashkent peripheral	159	152	(96)	5.29	(4.65,6.02)	4.69	(4.03,5.47)
Bukhara central	134	96	(72)	3.96	(3.36,4.67)	6.26	(5.12,7.66)
Bukhara peripheral	524	381	(73)	4.02	(3.51,4.60)	2.79	(2.43,3.19)
**Diagnostic Capacity**							
Microscopy and GeneXpert	555	22	(4)	Ref.		Ref.	
Microscopy only	943	454	(48)	12.15	(8.02,18.39)	7.75	(5.22,11.50)
No Microscopy no GeneXpert	442	356	(81)	20.32	(13.46,30.68)	6.31	(4.17,9.55)

* Distance from the health facility where the patient initially visited to the nearest GeneXpert laboratory; km = kilometers. RR = unadjusted relative risk; aRR = adjusted relative risk; CI = confidence intervals; Ref. = Reference group; Note: Factors with RR and 95% CI in bold are statistically significant (<0.05).

**Table 5:** Delays in Xpert MTB/RIF testing among presumptive tuberculosis patients in selected primary health centers (without Xpert testing services) of Tashkent City and Bukhara region of Uzbekistan, January–March 2019.

Duration (Days)	Number Eligible	Number Assessed	(%)	Median Days (IQR)	Max (Days)
**Total (Both the Regions)**					
Initial visit to PHC and sputum collection	1385	1368	(99)	0 (0,0)	8
Sample collection at PHC to receipt at GX laboratory	1368	576	(42)	1 (1,1)	13
Sample receipt at GX laboratory to result receipt at PHC	576	575	(99)	0 (0,0)	10
Total (Initial visit to PHC to receipt of result at PHC)	1385	575	(42)	1 (1,2)	18
**Tashkent City**					
Initial visit to PHC and sputum collection	830	813	(98)	0 (0,1)	8
Sample collection at PHC to receipt at GX laboratory	813	476	(59)	1 (1,1)	8
Sample receipt at GX laboratory to result receipt at PHC	476	475	(99)	0 (0,0)	1
Total (Initial visit to PHC to receipt of result at PHC)	830	475	(57)	1 (1,2)	9
**Bukhara Region**					
Initial visit to PHC and sputum collection	555	555	(100)	0 (0,0)	0
Sample collection at PHC to receipt at GX laboratory	555	100	(18)	2 (1,4)	13
Sample receipt at GX laboratory to result receipt at PHC	100	100	(100)	0 (0,4)	10
Total (Initial visit to PHC to receipt of result at PHC)	555	100	(18)	3 (1,6)	18

PHC = primary health centers; GX = GeneXpert; IQR = Interquartile range showing 25th and 75th centile; Max = maximum.

## Data Availability

The data that support the findings of this study are available from the corresponding author, (LT), upon reasonable request.

## Disclaimer

The authors alone are responsible for the views expressed in this publication and they do not necessarily represent the decisions or policies of the World Health Organization. ©World Health Organization 2021. All rights reserved. The WHO Regional Office for Europe has granted the Publisher permission for the reproduction of this article.

## Open Access Statement

In accordance with WHO’s open-access publication policy for all work funded by WHO or authored/co-authored by WHO staff members, the WHO retains the copyright of this publication through a Creative Commons Attribution IGO licence (http://creativecommons.org/licenses/by/3.0/igo/legalcode) (accessed on 18 February 2021) which permits unrestricted use, distribution and reproduction in any medium provided the original work is properly cited.
